# Supplementary material for: Content-rich biological network constructed by mining PubMed abstracts
Source: BMC Bioinformatics. 2004 Oct 8;5:147. doi: 10.1186/1471-2105-5-147 (PMC528731; doi:10.1186/1471-2105-5-147)
Supplement: Additional File 2 — The original results of the above study (non-essential files are deleted to keep the file size under the limit set by BMC bioinformatics). [file 1471-2105-5-147-S2.bz2 › chilibotAdditionalFile2/dip20/107ID1311321E442/html/MYOG_TCF3.html]

 


 **MYOG** and **TCF3** 
  
Found 23 abstracts in PubMed, retrieved 20.  
 

 What does Google say? 
 PDF only 
| .edu only 

---

**Interactive relationship** (e.g. stimulation, inhibition, etc)

**Neutral relationship**- Here, we report that Pbx Meis1 Prep1 binds DNA cooperatively with heterodimers of E2a  [ **TCF3** ]  and MyoD, myogenin  [ **MYOG** ] , Mrf 4 or Myf 5.  Ref: 10471746 Nucleic Acids Res, 1999
- We show that repression by TGF beta is directed at the basic HLH motif of myogenin  [ **MYOG** ]  and is independent of E2A  [ **TCF3** ]  products.  Ref: 1317849 J Biol Chem, 1992
- We show that dimerization with E2A  [ **TCF3** ]  products potentiates phosphorylation of myogenin  [ **MYOG** ]  at sites within its amino and carboxyl terminal transcription activation domains.  Ref: 8065355 Mol Cell Biol, 1994
- The ability of E2A  [ **TCF3** ]  products to potentiate myogenin  [ **MYOG** ]  phosphorylation suggests that dimerization induces a conformational change in myogenin  [ **MYOG** ]  that unmasks otherwise cryptic phosphorylation sites or that E2A  [ **TCF3** ]  proteins recruit a kinase for which myogenin  [ **MYOG** ]  is a substrate.  Ref: 8065355 Mol Cell Biol, 1994
- Phosphorylation of myogenin  [ **MYOG** ]  at these sites was mediated by the bHLH region of E2A  [ **TCF3** ]  products and was dependent on dimerization but not on DNA binding.  Ref: 8065355 Mol Cell Biol, 1994
- Repression of myogenin  [ **MYOG** ]  function by TGF beta 1 is targeted at the basic helix loop helix motif and is independent of E2A  [ **TCF3** ]  products.  Ref: 1317849 J Biol Chem, 1992
- In many instances tissue specific basic helix loop helix proteins, such as the hematopoietic factor SCL tal or the myogenic factor  [ **MYOG** ]  MyoD, interact with ubiquitously expressed basic helix loop helix proteins, such as E2A  [ **TCF3** ]  or E2 2.  Ref: 8576241 J Biol Chem, 1996
- In muscle, E2A  [ **TCF3** ]  proteins bind DNA as heterodimers with muscle specific bHLH partners, such as MyoD and myogenin  [ **MYOG** ] , and these heterodimers are thought to be both necessary and sufficient for muscle determination in cultured cells.  Ref: 7623842 Mol Cell Biol, 1995
- The muscle specific helix loop helix HLH proteins myogenin  [ **MYOG** ] , MyoD, myf5, and MRF4 form hetero oligomers with ubiquitous HLH proteins encoded by the E2A  [ **TCF3** ]  gene and activate muscle transcription by binding to a DNA sequence known as an E box CANNTG.  Ref: 1317849 J Biol Chem, 1992
- Myogenin  [ **MYOG** ]  is a muscle specific HLH protein that binds DNA in vitro as a heterodimer with several widely expressed HLH proteins, such as the E2A  [ **TCF3** ]  gene products E12 and E47.  Ref: 1325437 J Biol Chem, 1992
- The muscle specific basic helix loop helix bHLH protein myogenin  [ **MYOG** ]  activates muscle transcription by binding to target sequences in muscle specific promoters and enhancers as a heterodimer with ubiquitous bHLH proteins, such as the E2A  [ **TCF3** ]  gene products E12 and E47.  Ref: 8065355 Mol Cell Biol, 1994

**Non-interactive relationship** (e.g. studied together, co-existance, homology, etc.)

- In NIH3T3 fibroblasts, the ubiquitous helix loop helix HLH protein E2A  [ **TCF3** ]  E12 E47 and the myogenic HLH proteins MyoD, MRF4 and myogenin  [ **MYOG** ]  are growth inhibitory, while two ubiquitous Id proteins lacking the basic region are not.  Ref: 7925274 EMBO J, 1994
- Peptide growth factors, as well as the immediate early gene products c Jun, v Fos, and c Myc, inhibit the activity of myogenin  [ **MYOG** ]  through a mechanism independent of its association with E2A  [ **TCF3** ]  products.  Ref: 1325437 J Biol Chem, 1992
- Analysis of the oligomerization of myogenin  [ **MYOG** ]  and E2A  [ **TCF3** ]  products in vivo using a two hybrid assay system.  Ref: 1325437 J Biol Chem, 1992
- Mutation of this E box, which prevents binding of MyoD E2A  [ **TCF3** ]  and myogenin  [ **MYOG** ]  E2A  [ **TCF3** ]  heterodimers, decreases expression in myotubes and increases expression in myoblasts and nonmuscle cells.  Ref: 8355673 Mol Cell Biol, 1993
- An E box binding activity, which does not contain MyoD, myogenin  [ **MYOG** ] , or E2A  [ **TCF3** ]  proteins, is present in muscle and nonmuscle cells and may be responsible for repressing the delta subunit gene in myoblasts and nonmuscle cells.  Ref: 8355673 Mol Cell Biol, 1993
- Moreover, while ITF  [ **TCF3** ]  2b inhibited the creatine kinase promoter, it acted as a weak transactivator on an artificial promoter consisting of three tandem copies of the consensus myogenic factor  [ **MYOG** ]  DNA binding site.  Ref: 8999959 J Biol Chem, 1997
- The myogenic regulatory factors MRFs, MyoD, myogenin  [ **MYOG** ] , Myf 5, and MRF4, function as transcriptional activators of muscle specific gene expression by forming heterodimers with the ubiquitously expressed products of the E2A  [ **TCF3** ]  gene, E12 and E47.  Ref: 9116507 Protein Expr Purif, 1997
